# Design of the RINSE Trial: The Rapid Infusion of cold Normal Saline by paramedics during CPR

**DOI:** 10.1186/1471-227X-11-17

**Published:** 2011-10-13

**Authors:** Conor Deasy, Stephen Bernard, Peter Cameron, Ian Jacobs, Karen Smith, Cindy Hein, Hugh Grantham, Judith Finn

**Affiliations:** 1 Ambulance Victoria, Victoria, Australia; 2 Department of Epidemiology and Preventive Medicine Melbourne, Monash University, Australia; 3 Alfred Hospital, Melbourne, Australia; 4 Discipline of Emergency Medicine (M516), School of Primary, Aboriginal & Rural Health Care, University of Western Australia, Australia; 5 St John Ambulance, Belmont, Western Australia, Australia; 6 South Australia Ambulance Service, Eastwood, South Australia, Australia

## Abstract

**Background:**

The International Liaison Committee on Resuscitation (ILCOR) now recommends therapeutic hypothermia (TH) (33°C for 12-24 hours) as soon as possible for patients who remain comatose after resuscitation from shockable rhythm in out-of-hospital cardiac arrest and that it be considered for non shockable rhythms. The optimal timing of TH is still uncertain. Laboratory data have suggested that there is significantly decreased neurological injury if cooling is initiated during CPR. In addition, peri-arrest cooling may increase the rate of successful defibrillation. This study aims to determine whether paramedic cooling during CPR improves outcome compared standard treatment in patients who are being resuscitated from out-of-hospital cardiac arrest.

**Methods/Design:**

This paper describes the methodology for a definitive multi-centre, randomised, controlled trial of paramedic cooling during CPR compared with standard treatment. Paramedic cooling during CPR will be achieved using a rapid infusion of large volume (20-40 mL/kg to a maximum of 2 litres) ice-cold (4°C) normal saline.

The primary outcome measure is survival at hospital discharge. Secondary outcome measures are rates of return of spontaneous circulation, rate of survival to hospital admission, temperature on arrival at hospital, and 12 month quality of life of survivors.

**Discussion:**

This trial will test the effect of the administration of ice cold saline during CPR on survival outcomes. If this simple treatment is found to improve outcomes, it will have generalisability to prehospital services globally.

**Trial Registration:**

ClinicalTrials.gov: NCT01172678

## Background

Cardiovascular disease is a leading cause of premature death in Australia [1]. More than half of these deaths (approximately 25,000 per year) occur prior to hospital arrival. Despite sophisticated emergency medical service responses to sudden cardiac arrest, less than half of sudden cardiac arrest patients are able to be resuscitated by paramedics [2]. For those who are initially resuscitated and transported to hospital, the prognosis is still poor, particularly in rural areas [3]. Much of the mortality and morbidity after hospital admission is due to the anoxic brain injury sustained during the cardiac arrest [4].

One major recent advance in the treatment of severe anoxic brain injury following out-of-hospital cardiac arrest is therapeutic hypothermia (TH). When induced after resuscitation, this treatment was shown to improve neurological and overall outcomes in two randomized, controlled clinical trials [5,6]. The International Liaison Committee on Resuscitation (ILCOR) now recommends TH (33°C for 12-24 hours) as soon as possible for patients who remain comatose after resuscitation from out-of-hospital cardiac arrest for shockable rhythms and suggests that this therapy be considered for non shockable rhythms and in-hospital arrests [7,8].

The optimal timing of TH is still uncertain. Laboratory data have suggested that there is significantly decreased neurological injury if cooling is initiated during CPR [9-11]. Clinical and laboratory trials over the last three years have established that a rapid intravenous infusion of a large volume (20-40 mL/kg) of ice-cold fluid (i.e. normal saline) during CPR is a feasible and an effective method of induction of mild TH [12]. This technique has become widely used as a cooling method in the pre-hospital setting [13], the Emergency Department [14] and the Intensive Care Unit [15].

Previously, a randomised, controlled trial comparing paramedic cooling after return of spontaneous circulation (ROSC) with cooling in the emergency department was conducted in Melbourne. The study was stopped at the interim analysis due to a lack of difference in the primary outcome measure (outcome at hospital discharge) between the two groups [16]. Analysis of the data revealed that paramedics infused an average of 1000 mL ambient temperature saline during CPR prior to return of a spontaneous circulation as part of standard paramedic treatment and that cooling began approximately 30 minutes after paramedic arrival and only just prior to hospital cooling. Although there was a decrease in the core temperatures of the patients on arrival at the ED, this was a transient effect lasting only approximately 20 minutes. Subsequently, the cooling curves of the patients in both groups were identical. Thus, it was considered unlikely that this transient difference in core temperature could have a measurable effect on outcomes.

Further laboratory [17,18] and clinical research [19,20] has suggested that paramedic cooling during CPR is feasible and should be tested in large clinical trials. Boddicker et al [17] examined the success rates of defibrillation in swine cooled to different temperatures and found first-shock defibrillation success was highest in the hypothermia (33°C) group suggesting mild hypothermia may have a beneficial anti-arrhythmic effect, as well as a neuroprotective effect. Kämäräinen et al [20] cooled adult patients with out-of-hospital cardiac arrest during CPR and concluded that induction of therapeutic hypothermia during pre-hospital CPR was easily carried out and well tolerated. A study specifically examining respiratory function in patients treated with large volume, ice cold saline has indicated that there is no adverse effect on respiratory function [21] Garrett et al [22] in a retrospective analysis of a change in their prehospital cardiac arrest treatment protocols allowing intra-arrest induction of therapeutic hypothermia with 2000 ml of 4°C normal saline directly after obtaining IV/IO access concluded that TH during the intra-arrest period may improve the frequency of return of spontaneous circulation even at fluid volumes unlikely to change core body temperature.

Given these supportive laboratory and preliminary clinical data, we are conducting a definitive multi-centre, randomised, controlled trial of paramedic cooling during CPR compared with usual paramedic practice. We aim to determine whether paramedic cooling during CPR using a rapid infusion of large volume (20-40 mL/kg) ice-cold (4°C) normal saline improves outcome compared with standard treatment in patients who are being resuscitated from out-of-hospital cardiac arrest.

## Methods/Design

The RINSE trial is a prospective, multi-centre, randomised, single blinded, controlled trial conducted by the ambulance services of Victoria, Western Australia and South Australia.

In the treatment arm, paramedics will undertake immediate cooling on arrival and during cardiac arrest, using a large volume (20 mL/kg up to 2 litres) intravenous bolus of ice-cold saline. The saline infusion will be continued after return of circulation and en-route to hospital. In the control arm, patients will receive standard paramedic care, which includes the administration of normal saline at ambient temperature and will be cooled after arrival at the hospital (the current standard of care in ambulances in Australasia).

### Study sites

This is a three-centre funded study administered centrally through the Monash University Department of Epidemiology and Preventive Medicine with study sites in Victoria (Victoria Ambulance and Monash University); South Australia (South Australia Ambulance Service and Flinders University) and Western Australia (St John Ambulance and University of Western Australia). In Victoria, only MICA paramedics will enroll patients whereas in WA and SA all paramedics (but not transport officers) will recruit patients.

### Inclusion/Exclusion Criteria

Paramedics will screen patients during cardiac arrest and determine eligibility for enrolment. Adults 18 years and over, in cardiac arrest on arrival of paramedics are eligible for inclusion. Patients who are in cardiac arrest following trauma, or who are obviously pregnant or who are already hypothermic (tympanic temperature < 34.5°C) will be excluded.

### Randomisation

The ambulances will be provided with a set of randomisation envelopes. Block randomization will be used with instructions for immediate cooling therapy during CPR or instructions for standard treatment. Standard treatment includes cooling commenced at hospital as per ILCOR recommendations [7,8]. The envelopes will be randomised by computer-generated code into blocks of ten, numbered externally, and then sealed within an opaque envelope that conceals the treatment designation. All vehicles will carry two envelopes and as each is used, it will be replaced at the earliest convenient time from the remaining envelopes held at the ambulance station. Randomisation will be stratified by state to control for possible differences in paramedics skills and hospital treatment.

### Study Treatments

For patients randomised to paramedic cooling:

- Standard advanced cardiac life support

- Advanced Airway (Endotracheal Tube/Laryngeal Mask Airway) and ventilation with 100% oxygen

- Infuse 20 mL/kg cold fluid via IV stat during CPR

- Measure temperature using tympanic probe

- If temperature > 34.5°C, infuse further 10 mL/kg stat

- After ROSC, infuse further 10-20 mL/kg (to a maximum of 2 litres total fluid) ice-cold saline

- If shivering occurs post resuscitation, administer midazolam 2-5 mg IV and pancuronium 8 mg (Victoria only)

For patients randomised to hospital cooling:

- Standard advanced cardiac life support

- Advanced Airway (Endotracheal Tube/Laryngeal Mask Airway) and ventilation with 100% oxygen

- Measure temperature using tympanic probe

- Insert IV line and administer drugs as per protocol

- Fluid challenge with standard temperature saline only as per current patient care guideline

- Post resuscitation: midazolam 1-5 mg only to maintain intubation as needed

- Pancuronium 8 mg only if intubation unable to be maintained with midazolam (Victoria only)

After arrival at the Emergency Department, all patients receive standard care which may include cooling to 33°C for 24 hours.

### Cooled Fluid

The saline will be carried in insulated containers which are changed every shift. A thermometer is housed in this container ensuring the fluid is of the desired temperature.

### Sample Size

This study consists of two parallel clinical trials, separately testing the effect of paramedic cooling during CPR in patients with a shockable rhythm (VF/VT) and non-shockable rhythm (asystole/pulseless electrical activity).

The primary outcome measure for post-VF arrest patients is survival at hospital discharge. Data from the Victorian Cardiac Arrest Register shows that patients who are in ventricular fibrillation on arrival of paramedics have a 40% rate of return of spontaneous circulation, and there was a 50% subsequent survival rate in the previous Melbourne cooling trial [16]. The overall current survival rate based on all participating states is circa 20%. We propose that a rapid infusion of cold IV fluid will increase the rate of return of spontaneous circulation based on laboratory data cited above from 40% to 45%, and that this very early cooling will increase the overall survival rate from 20% to 27%. With 80% power and a type 1 error of 0.05, the study requires a sample size of 603 post VF-arrest patients in each arm (1206 in total).

Randomization of patients with non-VF will occur concurrently. The primary outcome measure for non-VF/VT cardiac arrest patients is also survival at hospital discharge. Currently, the outcome at hospital discharge of these patients is 2% [3]. To demonstrate improved outcomes to 5% (an absolute difference of 3%) requires 653 per group, a total of 1306 patients.

For both studies, secondary outcome measures are the rates of return of spontaneous circulation [23], survival to hospital admission on all patients, and quality of life measured by telephone follow up at 12 months using the Glasgow Outcome Scale Extended (GOSE) [24], EQ-5D [25] and SF-12 Health Survey Summary Score [26]. Analysis will be based on "Intention-to-treat".

### Consent/Ethics

Given that patients in cardiac arrest are unconscious, it is not possible to obtain informed consent prior to randomisation. The Australian National Statement on Ethical Conduct in Human Research [27] is used as the basis for ethical review across the three Australian states involved in this trial. Section 2.3.6 states that a human research ethics committee may grant waiver of consent if (a) involvement in the research carries no more than low risk, and (b) the benefits from the research justify any risks of harm associated with not seeking consent, and (c) it is impracticable to obtain consent and (d) there is no known or likely reason for thinking that participants would not have consented if they had been asked. Sections 4.4.13 requires there be no reason to believe that consent would not be forthcoming were it requested, that the risks of harm are minimized, that the project is not controversial and does not involve significant moral or cultural sensitivities in the community. Section 4.4.13 also requires that the research supports a reasonable possibility of benefit over standard care, that any risk or burden of the intervention to the participant is justified by its potential benefits and that inclusion in the research project is not contrary to the interests of the participant. Section 4.4.14 requires that as soon as reasonably possible, the participant and/or the participant's relatives should be informed of the participant's inclusion in the research and of the option to withdraw from it without any reduction in quality of care.

In addition, enrolment without consent is authorized in Victoria under Section 42A of the Medical Treatment Act. This states that a registered medical practitioner may carry out, or supervise the carrying out of, a medical research procedure on a patient without consent or authorisation if the practitioner believes on reasonable grounds that the treatment is necessary, as a matter of urgency (a) to save the patient's life; or (b) to prevent serious damage to the patient's health. This study meets the intent of this section of the Medical Treatment Act since the treatment (cooling during CPR) is a matter of urgency and has the intention of saving life and preventing serious neurological injury.

Human research ethics committees in the three study states have endorsed the study protocol In Victoria, the Human Research Ethics Committee requested that the Investigators send an explanatory letter to survivors to advise them of enrolment in the study. This letter will be sent about two months after the cardiac arrest to ensure that they have recovered sufficiently to understand the letter. For deceased patients, an explanatory letter will be sent to the next-of-kin also about two months after the cardiac arrest to ensure that they have recovered somewhat from the grief of the death of the relative. If a surviving patient objects to the collection of data, then no further data from that time onwards will be collected. Public engagement has been achieved using media news releases on the trial and information on the trial will also be provided to the public through ambulance service's subscriber newsletters and web sites.

In Western Australia, the University of Western Australia Human Research Ethics Committee requires that that surviving participants are informed of their participation as soon as practicable.

### Data Collection

All data relevant to the study is currently recorded by paramedics immediately after the case as a Patient Care Record. Furthermore, Victoria, Western Australia and South Australia each have a cardiac arrest registry that records the patient, event and outcome data consistent with the Utstein criteria [23] for all OHCAs attended. The Victorian Cardiac Arrest Registry currently conducts a 12-month quality of life follow-up on all survivors. Victorian survivors will therefore be contacted 12 months post cardiac arrest to complete a functional and quality of life outcomes telephone questionnaire.

### Data Safety Management

The Data Safety Monitoring Committee will undertake an interim analysis after 600 post VF and 600 non-VF patients have been enrolled in the study. The study will be stopped if there is a significant difference in the two arms (p < 0.001) at the interim analysis [28]. Given that the majority of patients die at the scene or in the hospital, and recurrent cardiac arrest at any time is possible with standard care, it is not considered appropriate to report every death to the Data Safety Monitoring Group as a serious adverse effect.

### Funding

The study has been funded by a project grant from the National Health and Medical Research Council (NHMRC) (grant number 1010613).

## Discussion

There are a number of factors that support the feasibility of this proposed trial. First, the Investigators have successfully undertaken a number of previous studies of therapeutic interventions in critically ill patients in the pre-hospital setting [16,29-32]. Second, the ambulance services in Victoria, Western Australia and South Australia are well placed to undertake large clinical trials. The paramedics in these states are highly trained and able to successfully enrol patients using a computer-generated envelope randomisation strategy, and then implement the required treatment. Third, the ethical issues associated with non-consent randomisation of unconscious patients have been carefully considered by Ethics Committees in each of the states. Our approach of delayed or non-consent has been accepted in the previous trials cited above. There is now a framework under the NHMRC that supports the conduct of such trials.

There will be important differences between the study protocol in Victoria, Western Australia and South Australia owing to variations in standard operating procedures and for this reason a stratified randomisation approach has been adopted. Ambulance Victoria has a different staffing structure whereby cardiac arrests are attended by intensive care paramedics with a wider scope of practice for airway intervention (intubation) and drug administration.

The Victorian ambulance protocol allows for endotracheal intubation while the South Australia and Western Australian protocols allow for the use of either endotracheal intubation or laryngeal mask airway. In addition, the Victorian ambulance protocol allows the administration of midazolam 2-5 mg IV and pancuronium 8 mg for sedation of patients in the post resuscitation phase as a treatment option for patient shivering. This will not be part of the Western Australia and South Australia protocol, thus we will be able to ascertain the prevalence of overt shivering following the infusion of cold fluids by the paramedics. Thus, we will be able to assess whether the suppression of shivering is important in the induction of therapeutic hypothermia by the measurement of temperature at hospital arrival.

There is an urgent need to improve outcomes from out-of-hospital cardiac arrest. The RINSE trial will test the effect of administration of a bolus of 20 mL/kg of ice cold saline during CPR on patient survival. If this simple, inexpensive treatment is found to improve patient outcomes it will be an important prehospital intervention globally.

## Competing interests

The authors declare that they have no competing interests.

## Authors' contributions

CD compiled this methodology paper, collaborated in the design of the trial, co-ordinated the initial ethics applications, co-ordinated the procurement of funding and paramedic education. SB is Chief Investigator, responsible for study design, governance, and roll out of the trial. PC is responsible for governance and logistical support as well as being a collaborator in the study design. IJ is responsible for governance, logistical, statistical support as well as being a collaborator in the design and in facilitating roll out of trial in South Australia and Western Australia. KS is responsible for governance, logistical and statistical support as well as a collaborator in the design. CH is responsible for governance, logistical, ethics application and trial roll out in South Australia. HG is responsible for governance, logistical support, and trial roll out in South Australia. JF is responsible for governance, ethics application, logistical, statistical support as well as collaborator in design and facilitating roll out of the trial in South Australia and Western Australia. All authors contributed substantially to the design and methodology of this study and to the writing and critical editing of this manuscript. SB, KS, PC, JF, IJ collaborated on procurement of funding for this trial. All authors have read and approved the final manuscript.

## Pre-publication history

The pre-publication history for this paper can be accessed here:

http://www.biomedcentral.com/1471-227X/11/17/prepub
